# Whole-Genome Sequences of Mycobacterium abscessus subsp. *massiliense* Isolates from Brazil

**DOI:** 10.1128/MRA.00361-21

**Published:** 2021-07-15

**Authors:** Edson Machado, Fernanda Cristina Oliveira, Rafael Silva Duarte, Ana Carolina Carvalho, Pedro Henrique Campanini Cândido, Emilyn Costa Conceição, Lia Gomes, Sidra E. G. Vasconcellos, Fabrice Santana Coelho, Solange Vinhas, Maria Cristina Lourenço, Ana Carolina R. Guimarães, Marcos Catanho, Philip Noel Suffys

**Affiliations:** a Laboratório de Genética Molecular de Microrganismos, Instituto Oswaldo Cruz, Fundação Oswaldo Cruz, Rio de Janeiro, Rio de Janeiro, Brazil; b Laboratório de Micobactérias, Departamento de Microbiologia Médica, Instituto de Microbiologia Paulo de Góes, Universidade Federal do Rio de Janeiro, Rio de Janeiro, Rio de Janeiro, Brazil; c Faculdade de Farmácia, Universidade Federal do Rio de Janeiro, Campus Macaé, Macaé, Rio de Janeiro, Brazil; d Laboratório de Bacteriologia e Bioensaios, Instituto Nacional de Infectologia, Fundação Oswaldo Cruz, Rio de Janeiro, Rio de Janeiro, Brazil; e Laboratório de Biologia Molecular Aplicada a Micobactérias, Instituto Oswaldo Cruz, Fundação Oswaldo Cruz, Rio de Janeiro, Rio de Janeiro, Brazil; f Hospital Universitário Pedro Ernesto, Universidade do Estado do Rio de Janeiro, Rio de Janeiro, Rio de Janeiro, Brazil; g Núcleo de Doenças Infecciosas da UFES, Universidade Federal do Espírito Santo, Vitória, Espírito Santo, Brazil; h Laboratório de Genômica Funcional e Bioinformática, Instituto Oswaldo Cruz, Fundação Oswaldo Cruz, Rio de Janeiro, Rio de Janeiro, Brazil; University of Maryland School of Medicine

## Abstract

The Mycobacterium abscessus complex comprises multidrug-resistant, opportunistic, and rapidly growing pathogens responsible for severe infections. Here, we report the genome composition of four Mycobacterium abscessus subsp. *massiliense* isolates from three sources: two from the lung of a cystic fibrosis patient, one from a mammary cyst, and one from a gutter system.

## ANNOUNCEMENT

The Mycobacterium abscessus complex (MABSC) is so far composed of three subspecies: Mycobacterium abscessus subsp. *massiliense*, Mycobacterium abscessus subsp. *bolletii*, and Mycobacterium abscessus subsp. *abscessus* (1). Although common in soil and water, they are frequent (opportunistic) human pathogens associated with a broad spectrum of diseases, ranging from pulmonary to superficial skin and soft tissue infections to severe disseminated infections in immunocompromised patients (2, 3), such as those with cystic fibrosis, among other chronic lung diseases (4).

Infections caused by the MABSC are becoming more prevalent worldwide (5), including in Brazil, which has reported 2,128 skin and soft tissue infections since 2004, with significant outbreaks (6). The effective treatment of infections caused by the MABSC is challenged by the high resistance to antibiotics displayed by these organisms (5, 7).

Here, we announce the genome sequencing of four clinical strains of M. abscessus subsp. *massiliense* (MAB1 to MAB4) isolated in Brazil. The samples were processed with *N*-acetyl-l-cysteine (NALC)-NaOH and 5% oxalic acid decontamination (8) and cultured in Lowenstein-Jensen medium in an incubator at 35°C for 7 days. All isolates were tested for susceptibility against amikacin (AMK), ciprofloxacin (CIP), doxycycline (DOX), tobramycin (TOB), clarithromycin (CLR), cefoxitin (CXT), moxifloxacin (MFX), linezolid (LZD), and trimethoprim-sulfamethoxazole (TMP-SXT) using the MIC protocol according to the Clinical and Laboratory Standards Institute (CLSI) (9) performance standards for susceptibility testing for rapidly growing mycobacteria (RGM).

MAB1 was isolated from a sewer wastewater station in the city of Vitoria, Espírito Santo State, in 2009 and demonstrated drug resistance to CIP, DOX, and TOB. MAB2 and MAB4 were isolated from sputum samples from the same patient with cystic fibrosis in November 2009 and May 2010, respectively. MAB2 was resistant to CIP, CLR, DOX, TMP-SXT, and MFX; MAB4 was resistant to the same drugs except that it showed susceptibility to CLR. The exact reason for this was not investigated but might be due to heteroresistance and isolation of a different fraction of the population on both occasions. Isolate MAB3 was from a biopsy specimen from a cyst that developed after implantation of a mammary prosthesis in 2003 and presented no drug resistance (Table 1).

**TABLE 1:** Genomic features and accession numbers of the Mycobacterium abscessus subsp. *massiliense* isolates sequenced

Isolate	Source	Drug resistance profile^*a*^	BioSample accession no.	GenBank accession no.	SRA accession no.	No. of reads	Genome size (bp)	Genome completeness (%)	No. of contigs	*N*_50_ (bp)	Genome coverage (×)	GC content (%)
MAB1	Sewer water	CIP (R, 4.0); DOX (R, 32.0); TOB (R, 16.0)	SAMN16557383	JADEYL000000000	SRS8489819	20,332,477	5,232,272	100.00	190	161,580	777	64.0
MAB2	Sputum	CIP (R, 4.0); DOX (R, >32.0); MFX (R, 4.0); CLR (R, >64.0); TMP-SXT (R, 4.0/76)	SAMN16557407	JADEYM000000000	SRS8489820	8,243,873	4,966,199	100.00	71	191,720	332	64.2
MAB3	Mammary cyst biopsy specimen	Pan-susceptible	SAMN16557408	JADEYN000000000	SRS8489821	1,658,365	5,051,696	99.05	654	14,176	66	64.1
MAB4	Sputum	CIP (R, 4.0); DOX (R, 32.0); MFX (R, 8.0); TMP-SXT (R, 4.0/76)	SAMN16557409	JADEYO000000000	SRS8489822	3,842,124	4,962,832	100.00	79	155,714	155	64.2

a R, drug resistance; CIP, ciprofloxacin; DOX, doxycycline; TOB, tobramycin; CLR, clarithromycin; MFX, moxifloxacin; TMP-SXT, trimethoprim-sulfamethoxazole.

Genomic DNA was obtained from the Lowenstein-Jensen culture using cetyltrimethylammonium bromide (CTAB) phenol-chloroform-based extraction and sequenced using the Nextera XT library preparation kit (Illumina, San Diego, CA, USA) on the Illumina HiSeq 2500 platform with 2 × 150-bp paired-end reads.

The raw reads were evaluated using FastQC v.0.11.9 (10) and filtered and trimmed using Trimmomatic v.0.36 (11) (LEADING:3, TRAILING:3, SLIDINGWINDOW:3:28, HEADCROP:19, and MINLEN:40). The genome sequences were *de novo* assembled using SPAdes v.3.11.1 (12), and the assembly quality was evaluated using Quast v.5.0.2 (13) and CheckM v.1.1.2 (14), applying the lineage analysis workflow. Annotation was performed using NCBI Prokaryotic Genome Annotation Pipeline (PGAP) v.4.3 (15). No plasmids were found in the four samples analyzed, according to the PlasmidSeeker v.1.3 tool (16). Default parameters were applied unless otherwise specified.

In summary, the genome size ranged from 4.96 to 5.23 Mb with samples harboring 4,932 to 5,469 genes, roughly representing a difference of 6% in size and 10% in gene content (Table 1). The environmental isolate displayed the largest genome sequence.

### Data availability.

The genomic sequences are publicly available in NCBI under the BioProject accession number PRJNA672126.
